# Functional Interplay between Cristae Biogenesis, Mitochondrial Dynamics and Mitochondrial DNA Integrity

**DOI:** 10.3390/ijms20174311

**Published:** 2019-09-03

**Authors:** Arun Kumar Kondadi, Ruchika Anand, Andreas S. Reichert

**Affiliations:** Institute of Biochemistry and Molecular Biology I, Medical Faculty, Heinrich Heine University Düsseldorf, 40225 Düsseldorf, Germany

**Keywords:** mtDNA, cristae, fusion and fission, mitochondrial dynamics, mitochondriopathy

## Abstract

Mitochondria are vital cellular organelles involved in a plethora of cellular processes such as energy conversion, calcium homeostasis, heme biogenesis, regulation of apoptosis and ROS reactive oxygen species (ROS) production. Although they are frequently depicted as static bean-shaped structures, our view has markedly changed over the past few decades as many studies have revealed a remarkable dynamicity of mitochondrial shapes and sizes both at the cellular and intra-mitochondrial levels. Aberrant changes in mitochondrial dynamics and cristae structure are associated with ageing and numerous human diseases (e.g., cancer, diabetes, various neurodegenerative diseases, types of neuro- and myopathies). Another unique feature of mitochondria is that they harbor their own genome, the mitochondrial DNA (mtDNA). MtDNA exists in several hundreds to thousands of copies per cell and is arranged and packaged in the mitochondrial matrix in structures termed mt-nucleoids. Many human diseases are mechanistically linked to mitochondrial dysfunction and alteration of the number and/or the integrity of mtDNA. In particular, several recent studies identified remarkable and partly unexpected links between mitochondrial structure, fusion and fission dynamics, and mtDNA. In this review, we will provide an overview about these recent insights and aim to clarify how mitochondrial dynamics, cristae ultrastructure and mtDNA structure influence each other and determine mitochondrial functions.

## 1. Mitochondrial Membrane Structure and Cristae Biogenesis

Mitochondrial shape is constantly adapting at the cellular and intra-mitochondrial levels in response to energetic and/or developmental cues. Mitochondria are enclosed by two membranes where the inner membrane (IM) characteristically folds inwards to form cristae. Cristae house the respiratory chain complexes and the F_1_F_O_ ATP synthase representing the major functional units for energy conversion. The rest of the IM, located parallel to the outer membrane (OM), is termed the inner boundary membrane (IBM). The current knowledge views the cristae membrane (CM) not just as mere invaginations of the IM into the matrix; instead, the CM is a structurally and functionally distinct subcompartment of the IM. This distinction is in part due to the recent rediscovery of crista junctions (CJs) with a diameter of 12–40 nm at the neck of cristae [1,2,3]. Based on this unique structure, CJs were proposed to restrict the normal passage of proteins, metabolites and protons both towards and away from cristae, thus separating the mitochondria into several subcompartments [4,5]. This implies that a mitochondrion has three aqueous subcompartments, namely, the intermembrane space (IMS) between the IM and OM, the intracristal space (ICS) and the matrix. 

The shape, size and number of cristae are constantly changing based on varying energy demands or other physiological changes. Almost half a century ago, Charles Hackenbrock reported ultrastructural alterations in mitochondrial cristae to occur upon metabolic changes [6,7]. This is classically described as the transition between orthodox (matrix expanded, state IV respiration, low ADP levels) and condensed (matrix compacted, state III respiration, high ADP levels) states of mitochondria, which were suggested to be critical for ATP production. Remodelling of cristae also occurs during apoptosis ensuring release of cytochrome *c* from the ICS to the cytosol resulting in a downstream cascade of caspase activation and cell death [8]. It is evident that aberrant and altered cristae are associated with several human diseases [3] but what are the molecular players required for cristae formation and maintenance? Albeit several factors are reported to modulate cristae morphology, we will first focus on three major protein complexes that are shown to be involved in the biogenesis of cristae and/or CJs: OPA1 (Optic Atrophy 1), F_1_F_O_ ATP synthase and the MICOS (Mitochondrial Contact Site and Cristae Organizing System) complex. 

### 1.1. Optic Atrophy Type 1 (OPA1)

OPA1 is a large dynamin-related GTPase present at the IM which plays a dual role in IM fusion as well as cristae biogenesis. Heterozygous mutations in *OPA1* cause optic atrophy associated with deafness and dementia [9,10,11], while a rare homozygous mutation is reported to be associated with early encephalomyopathy, cardiomyopathy and early death [12]. The regulation of OPA1 is complicated as it comprises 8 alternate splice variants and two proteolytic cleavage sites, which generate several forms of OPA1 broadly classified as long, IM-anchored forms (full length) or short, proteolytically cleaved forms [13,14]. Although the long forms of OPA1 are sufficient to manage its mitochondrial fusion activity [15], joint action of long and short OPA1 forms is proposed to be necessary to keep CJs closed, thereby limiting the diffusion/release of metabolites including cytochrome *c* [16,17]. The proposed role of s-OPA1 is to manage mitochondrial fission, cristae biogenesis or energetics [15,18,19]. Deletion of *Opa1* was characterized by the presence of balloon-like swollen cristae and widening of CJs modulating the release of cytochrome *c* to initiate apoptosis independent of its fusion activity [16]. How does OPA1 dually regulate IM fusion and cristae biogenesis? Based on elegant experiments, Walter Neupert and colleagues proposed recently that the formation of lamellar sheets of cristae is dependent on a preceding IM fusion event mediated by Mgm1, the baker’s yeast ortholog of OPA1 [20]. They suggest that post-OM fusion, the juxtaposed IM are tethered by Mgm1 to initiate the IM fusion along the IM-OM contact sites, which would generate a cristae-shaped sac protruding into the matrix. In contrast to this, tubular cristae are presumably formed by invaginations of IM. Similar and other models of cristae formation were discussed earlier [3], yet further experimental evidence supporting or excluding different models is urgently needed. 

### 1.2. F_1_F_O_ ATP Synthase

The F_1_F_O_ ATP synthase is best known for its essential role in generating ATP using energy stored in an electrochemical proton gradient across the IM. A rather novel role of this complex is to determine cristae ultrastructure. Monomeric F_1_F_O_ ATP synthase consists of F_1_ (catalytic) domain facing the matrix and F_O_ embedded in the CM. Although the monomeric enzyme is sufficient for ATP production, the F_1_F_O_ ATP synthase is able to form dimers and oligomers, consisting of rows of dimers, which not only control the stability of this multiprotein complex but also shape the IM and thus contribute to cristae formation. Formation of F_1_F_O_ ATP synthase dimers occurs via distinct interactions between adjacent F_O_ domains, e.g., via the dimer-specific subunits *e* and *g* or the subunit *ATP4* of this complex [21,22,23]. The stable association of two monomers at a defined angle leads to bending of the IM creating protrusions and a strong positive curvature at cristae tips [24,25,26,27,28]. Indeed, absence of dimerization of this complex by deleting dimer-specific subunits e or g results in perturbed cristae structure [22].

### 1.3. Mitochondrial Contact Site and Cristae Organizing System (MICOS) Complex

In baker’s yeast, Mic60/Fcj1 located at CJs was shown to be required for their formation [29]. Overexpression of Mic60/Fcj1 resulted in branched cristae resembling internal CJs not in close proximity to the OM. Mic60 is an important subunit of the MICOS complex; a large oligomeric complex present at CJs [30,31,32]. It is required for the formation of CJs and contact sites between the IM and OM and is highly conserved across phyla [33,34]. The various subunits of the MICOS complex are identified in yeast and mammalian model systems and are termed as MicX/MICX (with X indicating the approximate molecular weight) according to a uniform nomenclature proposed in 2014 [35]. Hitherto, seven main subunits of the MICOS complex have been identified in mammals, namely, MIC60/Mitofilin/Fcj1, MIC10/Minos1, MIC19/CHCHD3, MIC25/CHCHD6, MIC13/Qil1, MIC26/APOO and MIC27/APOOL [36,37]. Lack of individual subunits of MICOS complex results in loss or reduction of CJs and cristae detaching from the IBM forming concentric rings resembling onion-like slices in sections of electron micrographs [30,31,32]. The extent of the phenotype varies with the subunit deleted, so that they are classified as essential/core or peripheral components of the MICOS complex. MIC60 is considered as the pioneer of the MICOS complex as it is required for the formation of CJs and contact sites between IM and OM owing to its interaction with several OM proteins (TOM complex, SAM/TOB, SCL25A46, metaxin) [38,39,40,41,42,43,44]. Mic10 is considered the other core component as its deletion also leads to a virtual loss of CJs culminating in the appearance of cristae stacks appearing as onion-like slices. Mic10 is a small protein with a conserved glycine-motif in the transmembrane domain required for self-oligomerization and CJs formation [45,46,47]. Mic60 and Mic10 have the capability to bend membranes that control the formation of CJs [45,48,49]. MIC13/Qil1 is essential for formation of CJs in mammalian cells [50,51]. Loss of MIC13 causes a destabilization of MIC10, MIC26 and MIC27 while other subunits MIC60, MIC19 and MIC25 remain intact and can form a stable subcomplex. This indicates a hierarchy in the assembly pathway of the MICOS complex, suggesting two distinct subcomplexes, the MIC60-19-25 and the MIC10-13-26-27 subcomplex. MIC26 and MIC27 belong to the protein family of apolipoproteins and are deemed non-core components of the MICOS complex [52,53,54]. MIC27 can bind to cardiolipin, the signature lipid of mitochondria [53]. MIC26 exists in two forms: the non-glycosylated form is mitochondrially targeted and a subunit of the MICOS complex, while the glycosylated form is translocated to the ER and subsequently secreted [52]. Both MIC26 and MIC27 are required for maintenance of cristae morphology and respiration [52,53]. MIC19 and MIC25 on the other hand belong to CHCHD family of proteins. While the physiological role of MICOS is still not completely understood, depletion of its subunits affect several mitochondrial functions such as respiration, protein import, lipid transport, mtDNA organization, apoptosis, and autophagy. Altered levels of MICOS components are associated with a number of pathologies such as diabetes, cardiomyopathy, epilepsy, Down syndrome and Parkinson’s disease (PD) [3,55]. Mutations in *MIC13* are reported to cause severe forms of mitochondrial encephalopathy [56,57,58,59], and coding variants of *MIC60* present in the mitochondrial targeting sequence (MTS) are found in PD patients [60].

In addition to increasing number of studies revealing the individual roles of OPA1, the MICOS complex, and the F_1_F_O_ ATP synthase in determining cristae morphology, there is evidence these complexes functionally interact at different levels. In baker’s yeast, it was shown that Mic10 binds to oligomeric F_1_F_O_ ATP synthase and both Mic10 and Mic26 promote its oligomerization [61,62]. Mic60/Fcj1 play an antagonist role to subunits e and g of the F_1_F_O_ ATP synthase [29]. It was suggested that this interplay modulates the curvature of the IM locally near CJs and thereby regulates CJ formation. In addition, it was reported that OPA1 favors oligomerization of F_1_F_O_ ATP synthase to protect mitochondria from respiratory stress [63]. Moreover, OPA1 was suggested to function epistatic to the MICOS complex in order to regulate the width of CJs [42] and Opa1 was shown to physically interact with Mic60 [64]. Albeit Opa1 clearly affects CJs architecture, CJs can still be formed in the absence of Opa1 demonstrating that this complex is not absolutely required for formation of CJs *per se* [64]. Overall, it appears that OPA1 has an important regulatory role in determining cristae and CJ structure which acts in concert with the MICOS complex as well as the F_1_F_O_ ATP synthase. Interestingly, all three complexes are reported to affect the integrity and/or inheritance of mtDNA as discussed in the next sections.

## 2. Organization and Structure of mtDNA

Mitochondrial DNA (mtDNA) was initially visualized as fibrous structures within the mitochondria of developing chick embryo [65,66,67]. MtDNA was shown to be of universal occurrence across phyla ranging from protozoa to plants to higher vertebrates [68] possessing a circular topology of around 5 µm circumference in osmotically disrupted mitochondria [69]. Each mitochondrion released an average of two to six circular DNA molecules present as monomer or dimers either in extended or partially coiled configuration upon osmotic stress. In addition, 80% of mtDNA monomers remained bound to mitochondrial membranes [70]. It was later shown that mtDNA was associated to membrane-like structures near the D-loop or origin of mtDNA replication [71] which was later confirmed by the observation that mtDNA is located next to the IM when visualized by immunogold labelling using an anti-DNA antibody [72]. This was further corroborated by using correlative 3D super-resolution fluorescence iPALM imaging followed by FIB (focused ion beam)-SEM (Scanning electron microscopy) which showed that mtDNA is in close proximity with the CMs [73]. Discoveries leading to the identification of mtDNA as a separate entity paved the way for the sequencing of the entire human mitochondrial genome harboring 16,569 bases encoding 2 rRNAs, 22 tRNAs and 13 mRNAs [74]. MtDNA contains very few noncoding bases between these genes and even has overlapping genes accounting for high economy of mtDNA. MtDNA can lack fully encoded termination codons where the termination is completed post-transcriptionally with addition of the 3’-polyA tail, and contains exceptions from the universal genetic code accounting for its uniqueness [74]. The tRNA punctuation model was proposed in the early 1980’s according to which tRNA processing of primary transcripts, containing mRNAs and rRNAs, occurs at sites of interspersed tRNAs. Thus, tRNAs act as breaks or punctuations for genes encoding proteins or rRNAs [75,76]. This organization and its physiological consequences on translation are elegantly described elsewhere [77,78]. In addition, a comprehensive description of mtDNA replication and transcription in mitochondria has been well covered earlier [79,80]. Here, we summarize some necessary aspects in brief. The minimal mtDNA replisome consists of the helicase TWINKLE, which unwinds the mtDNA duplex template, with the mitochondrial single-stranded DNA-binding protein (mtSSB) stabilizing this state and allowing replication by DNA polymerase γ (POLγ). The transcription apparatus in mitochondria consists of a mitochondrial transcription factor A (TFAM), mitochondrial transcription factors B1 & B2 (TFB1M and TFB2M), mitochondrial transcription elongation factor (TEFM) and mitochondrial RNA polymerase (POLRMT) [79,80,81]. POLRMT cannot bind directly to promoter DNA and requires the assistance of TFAM and TFB2M. TFAM plays an important role in mitochondrial transcription by distorting mtDNA to induce negative supercoils [82] and forces the promoter DNA to undergo a U-turn on the mtDNA [83]. TEFM is present at the promoter before transcription begins and stimulates the activity of POLRMT besides assisting POLRMT to transcribe longer transcripts [79,80,81]. A major difference to how DNA is packed and organized in mitochondria as opposed to the nucleus is the absence of histones in mitochondria. Still, mtDNA is known to exist as foci within the mitochondrial matrix termed mt-nucleoids. Using confocal microscopy, the initial studies concluded that the number of mitochondrial nucleoids per cell ranged from 450 to 800 in cultured human cells with preliminary data showing an average ranging from 2 to 10 mtDNA copies per nucleoid [72,84]. The numbers of mtDNA copies per nucleoid and cell were 2–3 times higher in tumor cell lines compared to non-tumor cell lines such as fibroblasts. Nucleoids are dynamic structures and undergo fusion and fission dynamics, yet this is not thought to involve a major exchange of mtDNA content [72]. Indeed, when two heterologous mtDNA populations from different cells containing non-overlapping deletions were fused, fully functional protein complementation was achieved without exchanging mtDNA [85]. With the advent of diffraction-unlimited imaging techniques such as STED super-resolution microscopy, individual nucleoids marked as a single entity, limited by resolution of confocal microscopy, could be further resolved [86]. Hence, the average number of mt-nucleoids in a mitochondrion revealed by confocal microscopy was not represented accurately, as mitochondria imaged using STED super-resolution microscopy revealed roughly 1.6 times more nucleoids per cell. Consequently, the number of mtDNA molecules per nucleoid had to be revised to 1.4. The diameter of the mt-nucleoids has to be adapted from 250 nm to 100 nm in various cell types although a single mtDNA molecule has a contour length of around 5 µm. Several proteins distinct from nuclear histones help to pack mtDNA. TFAM (mitochondrial transcription factor A) is an essential protein interacting with mtDNA, playing an important role in transcription of mtDNA. Intriguingly, it possesses an additional role of packaging mtDNA. Increasing the ratio of TFAM molecules to number of mtDNA bases, from 1 TFAM molecule per 150 bp to 6 bp of mtDNA, progressively and consistently increased the mtDNA compaction of nucleoids [87]. This is mediated by the ability of TFAM molecules to bind across a DNA strand in a single mtDNA molecule as deciphered from in vitro reconstitution experiments using rotary shadowing electron microscopy (EM). In yeast, Abf2 is the mitochondrial DNA-packaging protein. Nucleoids undergo dynamic remodelling by forming either open or closed structures by modulating the ratio of Abf2 to mtDNA [88].

What is known about the in vivo role of TFAM? Murine whole body knockouts (KOs) of *Tfam* were lethal around embryonic day e8.5 to e10.5 showing that TFAM plays a critical role in embryogenesis [89]. MtDNA was absent at e8.5 in *Tfam* knockouts. In addition, KO embryos at e8.5 showed accumulation of enlarged mitochondria together with disorganized cristae and respiratory chain dysfunction as revealed by COX-SDH staining pattern [89]. A conditional knockout of *Tfam* in the heart and skeletal muscle using Ckmm-cre resulted in dilated cardiomyopathy where the animals succumbed between 2–4 weeks of age. At an ultrastructural level, EM of myocardium revealed enlarged mitochondria and vesicle-like cristae together with respiratory deficiency as observed in the whole body knockouts of *Tfam* [90]. In another conditional KO model, where *Tfam* was deleted exclusively in the skeletal muscle, mice suffered from myopathy and electron micrographs revealed enlarged mitochondria with abnormal cristae [91]. Conditional KO of *Tfam* in dopamine neurons causes reduced mtDNA expression associated with progressive neurodegeneration of dopamine neurons, impairment of motor function in adulthood and formation of intraneuronal inclusions containing mitochondria. These mitochondria exhibited abnormal mitochondrial membranes with vacuolization in some cases [92]. In fact, it was shown that ageing and PD were positively correlated with high levels of mtDNA deletions in neurons of the substantia nigra [93]. Substantia nigra sections of PD patients showed a decreased number of neurons which occupied less area characterized by the presence of classical Lewy bodies. Surprisingly, unhealthy mitochondria with deranged cristae and electron-dense deposits were also found in substantia nigra [94]. Mutations in *PINK1* also cause PD [95]. At a molecular level, it was recently identified that PINK1 phosphorylates MIC60 to stabilize its oligomerization placing MIC60 function downstream of PINK1 [60]. Intriguingly, some PD patients also displayed mutations in the MTS of MIC60 laying emphasis on the PINK1-MIC60 pathway. Overall, depletion of *Tfam* results in abnormalities in mitochondrial morphology, function, cristae organization and reduction in mtDNA level, pointing towards a complex interplay between them. In addition, whole body loss of TWINKLE also results in embryonic lethality at e8.5 [96]. Conditional KOs of *Twinkle* in the heart and skeletal muscle led to a decrease in the mtDNA content, mitochondrial transcripts and respiratory chain assembly. A homozygous knockin of the mutant Polγ mutator mouse model, which lacks its proof-reading activity, showed a 3 to 5 fold increase in the frequency of point mutations culminating in premature ageing, weight loss, hair loss, kyphosis, osteoporosis, and heart enlargement [97]. These studies indicate a functional link between PD, reduced mtDNA and occurrence of deformed cristae.

A variety of approaches have been used to decipher the mt-nucleoid proteome. (1) A considerable amount of proteins associated with mtDNA were discovered by classical coimmunoprecipitation (coIP), using antibodies against proteins already known to be present in mt-nucleoids (e.g., TFAM). Optimization of the methods of purifying mitochondrial nucleoids in Hela cells coupled with mild lysis using nonionic detergents and sedimentation followed by coIP using anti-TFAM and anti-mtSSB antibodies led to the identification of proteins interacting with mtDNA [98]. Coimmunoprecipitation with these two antibodies mostly yielded common proteins which were broadly classified into categories belonging to mtDNA replication and transcription, mtDNA binding and metabolism, mitochondrial chaperones and other miscellaneous proteins having a variety of other roles. (2) In a more stringent approach, formaldehyde-crosslinking was performed on mt-nucleoids in the presence of ionic detergents and high salt to release proteins non-covalently bound to mtDNA. The subset of proteins found in both studies included TFAM, mtSSB and other proteins involved in mtDNA replication and transcription. Interestingly, a nucleoid model consisting of core and peripheral layered structure was proposed. According to this, the inner core contained proteins obtained after formaldehyde-crosslinking and included proteins of mtDNA replication and transcription. The outer layer proteins obtained in native nucleoids included Hsp60, ATAD3 and prohibitins [99]. In fact, prohibitin 1 was proposed to maintain the stability of mtDNA copy number by regulating the TFAM levels [100]. ATAD3 has the capacity to bind to displacement loops. This interaction could be modulated leading to dissociation or association of mt-nucleoids [101]. (3) Recently, a proximity biotinylation assay, using Twinkle-APEX2 followed by mass spectrometry, was successfully used to decipher the nucleoid proteome [102]. Some novel proteins such as FASTKD1, C7ORF55 and NDUFS6 were found in addition to corroborating the known proteins present in nucleoids.

## 3. Interplay between Mitochondrial Dynamics and mtDNA

At the cellular level, mitochondria are arranged as an interconnected network with long and short tubules. Described as early as 1914 [103] and rediscovered later [104,105], mitochondria are highly dynamic organelles that constantly undergo fusion and fission events and move within cells. By using the power of yeast genetics, initial pioneering studies led to identification of several proteins localized both on the OM and IM responsible for fusion and fission [106,107,108]. In mammals, mitochondria undergo fusion with the help of large GTPases, Mitofusins 1 and 2 (Mfn1 and Mfn2) for the OM and OPA1 for the IM. Disruption of mitochondrial fusion results in fragmented mitochondria due to ongoing fission. Fusion is required for proper inheritance of mtDNA and content mixing, which helps to complement mitochondria, ensuring proper mitochondrial health [109,110]. For mitochondrial fusion, the outer membranes of apposing mitochondria are first tethered and juxtaposed via homo- or hetero-oligomerization of MFN1 and MFN2, and after GTP hydrolysis, a conformational change needed for mitochondrial fusion is induced [111,112,113,114]. When isolated mitochondria were subjected to classical protease treatment followed by western blotting using antibodies specific for certain domains of mitofusins, it was deciphered that mitofusins harbor two transmembrane segments [115]. However, recently it was also suggested that the helical repeats HR2 are present in IMS and that mitofusins only harbor one transmembrane segment [116,117]. Mutations in *MFN2* are linked to Charcot-Marie-Tooth neuropathy type 2A (CMT2A) [118]. Fission in mammals is coordinated by another large dynamin-like GTPase, namely, DRP1. DNM2, MFF (mitochondrial fission factor), MID49, MID51 (mitochondrial dynamics proteins of 49 and 51 kDa) and FIS1 (mitochondrial fission 1 protein) are other factors which play a role in mitochondrial fission [119,120,121,122,123,124,125,126]. Loss of mitochondrial fission results in hyperfused mitochondria due to ongoing fusion. Mitochondrial fission has been proposed to ensure proper transport of mitochondria (e.g., in neurons) and to promote apoptosis [110,127]. Mitochondrial dysfunction results in alteration of mitochondrial dynamics and, combined with the selective removal of such damaged mitochondria by mitophagy, is critical for mitochondrial quality control [128,129,130,131,132]. DRP1 is recruited to mitochondria by several adaptors present at OM of mitochondria, namely, MFF, MID49, MID51 and FIS1 [133,134,135]. DRP1 causes mitochondrial constriction but DYN2 was proposed to work in collaboration with DRP1 to perform the final scission of the membranes [136]. However, more recent studies showed that all three dynamin proteins including DYN2 (DNM2) are dispensable for mitochondrial and peroxisomal fission [137,138]. DRP1 was shown to possess constricting as well as severing ability [137]. Apart from these molecules, ER contact with mitochondria and actin cytoskeleton also play crucial roles in mitochondria division [139,140]. Mutation in *DRP1* leads to manifestation of severe defects and early death including childhood epileptic encephalopathy, microcephaly and optic atrophy [141,142,143].

Next we focus on the question ‘Why does alteration of mitochondrial dynamics result in dual problems, namely, aberrant cristae and loss of mtDNA and/or aberrancies in mt-nucleoid morphology?’ Initial observations in yeast showed that deletion of the fusion factors Fzo1 and Mgm1, located on the OM and IM, respectively, caused loss of mtDNA [144,145,146,147]. Similarly, deletion of *Ugo1* which physically links Fzo1 and Mgm1 also resulted in mtDNA loss [148,149] providing evidence that defective fusion leads to loss of mtDNA. *Mfn1* and *Mfn2* null mice were never obtained with embryonic lethality occurring at midgestation around E12 stage [120]. It was shown that mtDNA levels were not altered in *Mfn* (Double Knockout) DKO mice although the mitochondrial fusion was strongly abolished resulting in fragmented mitochondria. It is possible that *Mfn* DKO mice exhibit lethality around the time mtDNA levels begin to drop, providing an alternate explanation for detecting no change in mtDNA when mitochondrial OM fusion is inhibited. Embryonic lethality of *Mfn2* null mice encouraged researchers to study conditional KOs of *Mfns* in various tissues. Intriguingly, loss of *Mfn2* in the cerebellum resulted in Purkinje cells (PCs) with swollen mitochondria having abnormal vesicular cristae [150]. These mice show growth defects, problems in limb coordination, and difficulties in gaining posture after being placed on their back and mostly move by writhing using abdomen as support. Also, depletion of Mfn1, Mfn2, Opa1 or Mfn1 & Mfn2 together in MEFs clearly showed loss of mitochondrial nucleoids when compared to control MEFs [150] which could have accounted for loss of membrane potential, decreased growth rates, and reduced mitochondrial oxygen consumption [151]. In accordance with the deletion of *Mfns* in cerebellum, where swollen mitochondria with sparse cristae were observed, loss of *Mfn1* & *2* in skeletal muscle led to both interfibrillar and subsarcolemmal fragmented mitochondria containing swollen and sparse cristae [152]. Skeletal muscle specific KOs of *Mfns* had growth defects when compared to control littermates and displayed reduced levels of non-fasting and fasting blood glucose levels when compared to controls pointing to metabolic aberrations when the ultrastructural integrity of mitochondrial cristae is compromised. As a result, respiratory function was defective indicated by skeletal muscle COX/SDH staining revealing a low COX activity and an increased SDH activity due to mtDNA defects. Intriguingly, these mice also show a drastic decrease of mtDNA copies per nuclear genome, strongly indicating that the integrity of cristae and mtDNA are interlinked. *Mfn2* DKO in the adult hearts results in progressive dilated cardiomyopathy [153]. Here, the cardiomyocytes displayed mitochondrial fragmentation and abnormal cristae morphology coupled with respiratory deficiency highlighting the intricate relationship between mitochondrial dynamics, cristae and respiratory efficiency. More recently, mitochondrial fusion was shown to be necessary for maintaining mtDNA replication and distribution [154]. *Mfn2* DKO in the heart resulted in higher heart to body weight ratio, indicating hypertrophy and abnormal mitochondria with disrupted ultrastructure in the form of irregularly arranged swollen cristae. MtDNA content was reduced in *Mfn* DKO in the heart but there were no adverse effects on the number of breakpoints in mtDNA or other signs of impaired mtDNA integrity. In addition, the frequency of mtDNA mutations was not altered. STED super-resolution microscopy of mt-nucleoids revealed that deletion of *Mfns* and *Opa1* led to mt-nucleoid clustering without affecting the size of mt-nucleoids [154]. Loss of fusion further resulted in a reduction of the steady-state levels of mtSSB, whereas Polγ levels and TWINKLE levels were increased indicating alteration of major proteins possibly compromising formation of the mtDNA replisome. This could explain decreased mtDNA copy numbers upon loss of mitochondrial fusion. Despite this, no impairment in mtDNA transcription was observed when mitochondrial fusion was impaired compared to controls based on in organello transcription experiments. Overall, loss of mitochondrial fusion due to loss of OM fusion leads to aberrations in mtDNA replication accompanied by defects in mitochondrial cristae organization [154].

How does a loss of mitochondrial fission lead to abnormalities in mt-nucleoid organization and to problems in cristae biogenesis? DRP1 plays an important role in the fission of the OM as discussed before. The physiological role of DRP1 is evident as mice deleted for *Drp1* exhibit embryonic lethality by midgestation where KO embryos were smaller than controls [155,156]. Analysis of mitochondrial ultrastructure by EM mostly showed no change in the internal organization of the interconnected large mitochondrial network [156]. Concurrently, no changes were observed in mtDNA levels of *Drp1* KO MEFS [155]. In contrast, another study reported loss of mtDNA, reduced membrane potential and cellular ATP levels in cells deleted for *DRP1* [157]. Drp1 was further shown to be required for the maintenance of PCs, and mice deficient in Drp1 exhibited reduced latency when a rotarod test was used to test motor coordination ability [158]. Here, loss of Drp1 in postmitotic neurons led to formation of swollen mitochondria, respiratory impairment and enhanced ubiquitination of mitochondria colocalizing with LC3 punctae, suggesting induction of mitophagy in PCs. Isolated cerebellar neurons deficient for Drp1 in culture faithfully mimicked mitochondrial swelling and cell death. In addition, *N*-acetylcysteine (NAC) treatment obliterated the mitochondrial swelling phenotype of *Drp1* KO PCs in culture indicating that oxidative damage promotes formation of swollen mitochondria. Thus, it could be that oxidative damage disrupts mtDNA and associated proteins forming nucleoids which may result in swelling of mitochondria and alteration of cristae morphology. In fact, depletion of *Drp1* in MEFs led to formation of huge nucleoids in mitochondria [159]. In this study, it was proposed that clustering of nucleoids led to reorganization of cristae into densely packed units called mito-bulbs. Interestingly, in 96% of the cases mt-nucleoids were located next to Drp1 and Mff spots, players in IM fission. Mt-nucleoid clustering was apparently upstream of mito-bulb formation because inhibition of mtDNA replication by 2’-3’-dideoxycytidine (ddC) treatment, leading to loss of nucleoids, followed by depletion of Drp1 did not lead to formation of mito-bulbs. In addition, nucleoid clustering paired with mito-bulb formation led to a delay of cytochrome *c* release and apoptosis. Overall, it appears that in this context mito-bulb formation is a consequence of formation of enlarged mt-nucleoids. Death of heart and skeletal muscle specific KOs of *Drp1* mice occurred within 11 days of birth, which was ascribed to dilated heart exhibiting hypertrophy and aberrant parameters deciphered by echocardiography. *Drp1* KO heart exhibited enlarged mitochondria with respiratory deficiency, nucleoid clustering and densely packed cristae compared to controls [160]. Hence, loss of mitochondrial fission apparatus leads to aberrant mt-nucleoids and associated disruption of cristae in heart. Mice deficient in Mff, a protein required for mitochondrial fission, in the whole body, die around 13 weeks of age with symptoms of dilated cardiomyopathy associated with heart failure. There is a decrease of mtDNA molecules with age coinciding with abnormal internal structure of mitochondria containing vacuoles. Additionally, there is reduced mitochondrial oxygen consumption, increased LC3 and p62 punctae co-localizing with ubiquitinated mitochondria suggesting increased mitophagy. Knockout of *Mfn1* in combination with *Mff* led to complete rescue of lifespan by rescuing the cardiac defects together with rescuing the mitochondrial oxygen consumption and autophagy. Intriguingly, the mtDNA levels were increased in DKO of *Mff* and *Mfn1* reiterating that balanced mitochondrial dynamics maintain mtDNA at steady state [161]. This is reminiscent of data from baker’s yeast showing that strains lacking Dnm1 and Fzo1 (or Dnm1 and Mgm1) are rescued from mtDNA loss [162,163,164] strongly indicating that lack of mitochondrial fusion only led to loss of mtDNA when mitochondrial fission is functional. Loss of both processes, however, will prevent content mixing which was shown to ensure complementation of mitochondria containing different mutant mtDNA molecules. This was exemplified earlier as polykaryons formed upon PEG fusion of wild type (WT) cells and cells devoid of mtDNA (rho^0^ cells) resulted in the distribution of mitochondrial nucleoids from WT mitochondria to rho^0^ mitochondria [84]. Moreover, mtDNA synthesis was shown to couple with mitochondrial fission at the ER–mitochondria contact sites [165]. Overall, several studies support the view that impairment of mitochondrial dynamics can result in mitochondrial dysfunction often linked to loss of mtDNA, accumulation of enlarged/altered mt-nucleoids and altered cristae morphology. To decipher these interdependencies in more detail, we will now focus on the impact of cristae biogenesis on mtDNA integrity and vice versa.

## 4. Interplay between Cristae Biogenesis and mtDNA

We have introduced the roles of three factors on cristae biogenesis, namely, OPA1, the MICOS complex and the F_1_F_O_ ATP synthase. How do these factors influence mtDNA organization? Mutations in *OPA1* cause Autosomal Dominant Optic Atrophy (ADOA) characterized by optic nerve atrophy leading to progressive loss of vision [9,11]. Patients suffering from autosomal dominant optic atrophy were found to contain reduced mtDNA content [166]. Hela cells downregulated for *OPA1* displayed swollen cristae and lower membrane potential than control cells [17]. *Opa1*^+/−^ mice showed a delayed phenotype of abnormal cardiac function resulting in decreased cardiac output coinciding with the onset of blindness. Strikingly, the *Opa1* mutant hearts also displayed loss of cristae coupled with impaired respiratory function of ETC complexes I, II and IV and reduction in mtDNA copy number [167]. Similarly, another study used a mouse model containing a splice site mutation leading to 27 amino acid residues’ deletion in the GTPase domain [168]. This led to 50% production of full length OPA1 protein where heterozygous mutant mice developed an age dependent loss of retinal ganglion cells (RGCs) and axons of the optic nerve displayed disorganized cristae in *Opa1* mutant mice. Consistent with this, another study showed that heterozygous OPA1^+/−^ mice showed an increase in the number of mitochondria harboring swollen cristae in the optic nerve and the hippocampus but did not show any change in the number of CJs [64]. A muscle specific deletion of *Opa1* led to death of all *Opa1* knockouts by postnatal day 9 (P9) corroborating its importance in early development of the mice [169]. Therefore, a tamoxifen-induced deletion of *Opa1* was performed at 5 months of age, which resulted in muscle atrophy, weakness, kyphosis and hair greying. Mitochondria of *Opa1* KO mice were smaller compared to controls and had dilated cristae. Since mtDNA depletion was not observed, the authors proposed loss of mitochondrial fusion upon *Opa1* deletion leads to irregularities in cristae shape and mitochondrial supercomplex assembly [169]. This was in line with a study reporting that mtDNA depletion results from chronic fusion inhibition [170]. In the latter study, acute Opa1 ablation was performed using *Opa1* floxed MAFs transduced with Cre. During acute ablation of Opa1 cristae shape and respiratory chain complexes were already impaired, while the mtDNA levels remained normal suggesting that cristae shape defects are not necessarily linked to mtDNA loss. Depletion of OPA1 and DRP1 simultaneously in the skeletal muscle of mice did not rescue accumulation of dysfunctional mitochondria where one-third of the mitochondria possessed either abnormal size or disrupted cristae structures supporting the view that a balance of mitochondrial fission and fusion is very important for regulation of mitochondrial size and cristae structure. Muscle weakness and atrophy were not rescued but a reduction of oxidative stress, denervation and FGF21 induction, which contributes to muscle atrophy, was observed [171]. 

Loss of MIC60 leads to loss of CJs, physically separating the IBM from the CM, and the appearance of concentric stacks of CM [29,172]. Depletion of MIC60 in both yeast and mammalian cells led to formation of enlarged nucleoids [173,174] demonstrating a rather direct link of altered cristae morphogenesis on mtDNA organization. Depletion of MIC60 in mammalian cells led to formation of enlarged mitochondria where mitochondrial fusion and fission events were reduced [174]. This was coupled to the formation of enlarged nucleoids and a drastic decrease of transcription of mt-encoded genes. This led to the hypothesis that CJs could be responsible for the distribution of nucleoids within the mitochondrial matrix in manner that is partially dependent on DRP1 [174]. In line with the antagonistic roles of Mic60/Fcj1 and dimer-specific subunits e and g of the F_1_F_O_ ATP synthase [29] it was shown that in yeast enlarged mt-nucleoid size was prevented when both, Mic60/Fcj1 and subunits e (or g) were deleted simultaneously [173]. Depletion of MIC10 did not induce formation of enlarged nucleoids while deletion of *MIC19* resulted in enlarged nucleoids. Also, deletion of Mic60/Fcj1 or subunits e (or g) alone was reported to cause a partial loss of mtDNA [29,175]. In addition, absence of mitochondrial fission by *Dnm1* deletion led to the formation of larger nucleoids in DKO strain of *Mic60* and *Dnm1* compared to *Mic60* KO showing that Dnm1 participated to distribute nucleoids [173]. 

Diabetic cardiomyopathy is a condition where patients suffering from type I diabetes are prone to increased risk of heart failure. Mic60 is significantly reduced in the interfibrillar mitochondria (IFM) of mice hearts which were insulted with streptozotocin to induce diabetes [176]. Accordingly, a *Mic60* knockin mice model was used to check whether cardiomyopathy induced by diabetes could be rescued in this genetic background [177]. Cardiac contractile function was reduced in diabetic mice which was rescued in diabetic mice overexpressing *Mic60* pointing out a beneficial role of high amounts of Mic60 under diabetic conditions. Interestingly, the cristae morphology altered in the IFM under diabetic conditions was rescued in *Mic60* knockin mice. This further points to an important role of cristae remodelling in the development and progression of diabetic cardiomyopathy [177]. The role of mtDNA mutations in cardiac pathologies is well documented [178]. Patients harboring pathological mtDNA mutations displayed swollen mitochondria, and mitochondria with concentric cristae once again corroborating the interplay between mtDNA and mitochondrial cristae in the context of cardiac pathology [179]. Taken together, remodelling of cristae appears to have an immediate impact on mt-nucleoid structure and inheritance. Further analysis of the effect of cristae shaping proteins on the organization of mt-nucleoids is needed. A summary of deletion of factors playing a role in mitochondrial dynamics (and cristae organization) leading to aberrations in nucleoids/mtDNA content and cristae organization is provided in Table 1.

## 5. Conclusions

**Figure 1:**
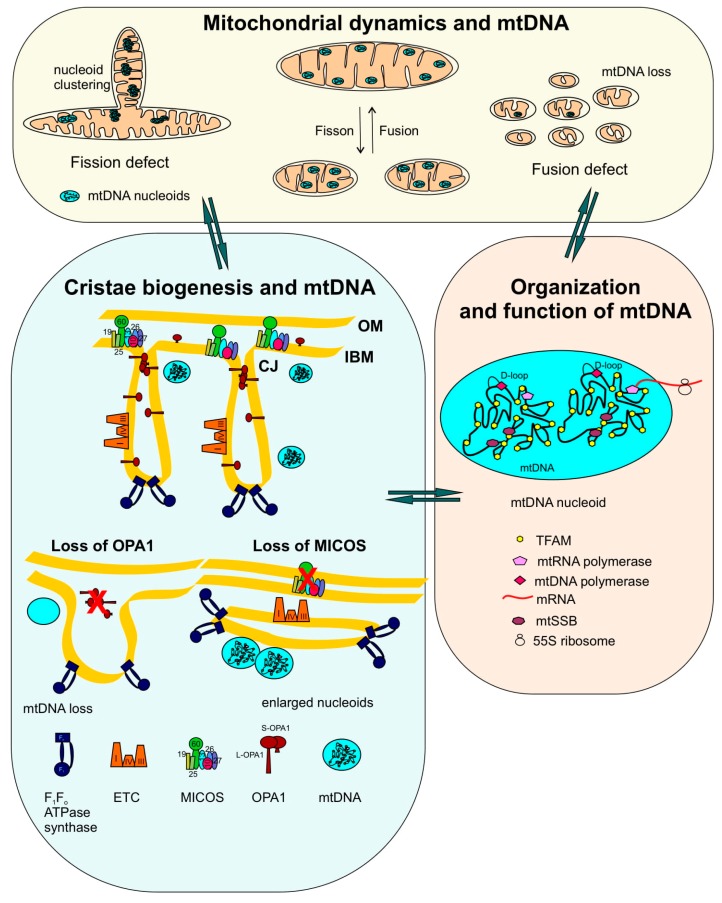
Functional interplay of mitochondrial dynamics, cristae biogenesis and mtDNA integrity: The scheme describes that the balance between mitochondrial dynamics, cristae biogenesis and mtDNA structure helps to manage integrity and function of mitochondrial DNA. Mitochondrial dynamics comprising fission and fusion cycles helps in redistribution and complementation of mtDNA within mitochondria. Lack of fusion causes subsequent loss of mtDNA from the fragmented mitochondria, whereas accumulation of aggregated nucleoids is observed in elongated mitochondria generated during a fission defect. Relationship between mitochondrial dynamics and cristae biogenesis and mtDNA organization is evident by several examples where the loss of fusion or fission concurrently shows defective cristae and mtDNA loss. Altered morphology of mitochondria is observed with mtDNA aberrations during cristae biogenesis defects. The internal mitochondrial structure is modulated by the following key players regulating cristae shape: OPA1 oligomers, MICOS, and F_1_F_o_ ATPase synthase. Chronic loss of OPA1 is accompanied by cristae defects as well as mtDNA loss. Depletion of MIC60 results in loss of CJs together with accumulation of enlarged nucleoids. The function and organization of mtDNA nucleoids is schematically depicted. On average, 1.4 mtDNA molecules are present per nucleoid which also contains associated proteins required for compaction, replication and transcription. ETC, electron transport chain (complex II is not shown).

We have tried to summarize a rather complex and interdependent interplay between mitochondrial dynamics, cristae biogenesis and mtDNA maintenance and integrity (Figure 1). It is a challenging and open question whether abnormalities in mitochondrial dynamics and cristae structure give rise to aberrancies in mtDNA and nucleoids or vice-versa. Based on the data available, the answer to this puzzle is still not clear—also, each case might have a different mode of action and it is possible that these processes are interdependent and cannot be separated that easily (as shown in Figure 1). It is not always that a reduction in mtDNA copy number, variations in nucleoid size and associated clustering are associated with disorganized cristae or the other way round. Also, alteration of mitochondrial dynamics has distinct effects on cristae and mtDNA integrity. At least we can say that loss of mitochondrial dynamics frequently leads to aberrant cristae formation and that aberrations in cristae morphology frequently result in loss of mtDNA. It is obvious that these processes are interlinked at multiple levels. Future studies will have to dissect the molecular hierarchy and interdependency of these processes. This will have important implications for understanding human diseases linked to various forms of mitochondrial dysfunction.

## Figures and Tables

**Table 1:** Summary of phenotypes linked to deletion of factors involved in mitochondrial dynamics or cristae biogenesis.

#	Gene-Specific Depletion	Gene Function	Model System	Phenotype	Cristae Abnormalities	Mitochondrial DNA/Nucleoid Aberrancy	Reference #
1	*Mfn1* & *Mfn2* DKO	OM fusion	Whole body KO in mice	Embryonic lethality	Not checked in mice	No change in mutant cell lines in mtDNA levels	[120]
2	*Mfn2* KO	OM fusion	Conditional KO in PCs of mice	Growth defects, limb coordination and posture problem	Sparse cristae with swollen mitochondria	Not analyzed in PCs	[150]
3	*Mfn1* KO, *Mfn2* KO, *Mfn1* and *Mfn2* DKO	OM fusion	Mammalian cell lines	Decreased growth rate, reduced oxygen consumption, membrane potential lost	Not checked in cells	Loss of mtDNA in Mfn1 and Mfn2 DKO and loss of nucleoids in single and double KOs	[150,151,152]
4	*Mfn1* & *2* DKO	OM fusion	Conditional KO in skeletal muscle	Growth defects and defunct metabolic homeostasis like reduced fasting and nonfasting blood glucose levels	Sparse cristae with swollen, fragmented mitochondria with less cox activity	mtDNA copy number reduced	[152]
5	*Mfn1* & *2* DKO	OM fusion	Conditional KO in heart (and skeletal muscle)	Dilated cardiomyopathy and Cardiac hypertrophy in ref. 153 & 154 respectively	Irregular arrangement of abnormal cristae	mtDNA content reduced, nucleoid clustering in ref. 154	[153,154]
6	*Drp1* KO	OM fission	Whole body KO in mice	Embryonic lethality	No change	No change	[155,156]
7	*Drp1* KO	OM fission	Conditional KO in PCs	Death of PCs and motor coordination ability compromised	Not checked	Not checked	[158]
8	*Drp1* KO	OM fission	Mammalian cell lines	Apoptosis delayed	Densely packed cristae termed mito-bulbs	Nucleoid clustering	[159]
9	*Drp1* KO	OM fission	Conditional KO in heart and skeletal muscle	Mice die by P10, Dilated heart due to hypertrophy	Densely packed cristae	Nucleoid clustering	[160]
10	*Drp1* KO	OM fission	Mammalian cell lines	Membrane potential reduced, ATP levels reduced	Not checked	mtDNA loss	[157]
11	*Opa1* KO	IM fusion	Mammalian cell lines	membrane potential reduced	Disorganised cristae	Not checked	[17]
12	*Opa1* KO	IM fusion	OPA1, heterozygous mice	Decreased cardiac output, onset of blindness	Cristae loss	mtDNA loss	[167]
13	*Opa1* KO	IM fusion	Inducible conditional deletion in skeletal muscle	Reduced body weight, muscle atrophy and weakness, kyphosis and hair greying	Dilated cristae	mtDNA unchanged	[169]
14	*Opa1* KO	IM fusion	*OPA1* heterozygous mice	Not reported	Swollen cristae in hippocampus and optical nerve; number of CJs unaltered	Not checked	[64]
15	*Mff*	OM fission	Whole body KO	Premature death at 13 weeks of age, heart failure due to cardiomyopathy	Vacuolated mitochondria with disorganised cristae	Reduced mtDNA	[161]
16	*Mic60*	CJs formation and assembly	Mammalian cell lines	Reduced mitochondrial dynamics	Loss of CJs leading to cristae separated from the IBM	Enlarged nucleoids, decrease in transcription of mitochondrial-encoded genes	[174]

(1) Mfn1 & 2: Mitofusins 1 & 2, (2) DRP-1: Dynamin-related Protein 1, (3) OPA1: Optic Atrophy Type 1, (4) MFF: Mitochondrial Fission Factor, (5) OM: Outer Membrane, (6) IM: Inner Membrane, (7) CJs: Crista Junctions, (8) KO: Knock out, (9) P10: Postnatal day 10, (10) PCs: Purkinje Cells.
